# Context and prediction matter for the interpretation of social interactions across species

**DOI:** 10.1371/journal.pone.0277783

**Published:** 2022-12-07

**Authors:** Theresa Epperlein, Gyula Kovacs, Linda S. Oña, Federica Amici, Juliane Bräuer

**Affiliations:** 1 DogStudies, Max Planck Institute for Geoanthropology, Jena, Germany; 2 Department for General Psychology and Cognitive Neuroscience, Friedrich Schiller University of Jena, Jena, Germany; 3 Department of Biological Psychology and Cognitive Neuroscience, Friedrich Schiller University of Jena, Jena, Germany; 4 Max Planck Research Group Naturalistic Social Cognition, Max Planck Institute for Human Development, Berlin, Germany; 5 Department of Comparative Cultural Psychology, Max-Planck Institute for Evolutionary Anthropology, Leipzig, Germany; 6 Behavioral Ecology Research Group, Institute of Biology, Faculty of Life Science, University of Leipzig, Leipzig, Germany; University of Lincoln, UNITED KINGDOM

## Abstract

Predictions about others’ future actions are crucial during social interactions, in order to react optimally. Another way to assess such interactions is to define the social context of the situations explicitly and categorize them according to their affective content. Here we investigate how humans assess aggressive, playful and neutral interactions between members of three species: human children, dogs and macaques. We presented human participants with short video clips of real-life interactions of dyads of the three species and asked them either to categorize the context of the situation or to predict the outcome of the observed interaction. Participants performed above chance level in assessing social situations in humans, in dogs and in monkeys. How accurately participants predicted and categorized the situations depended both on the species and on the context. Contrary to our hypothesis, participants were not better at assessing aggressive situations than playful or neutral situations. Importantly, participants performed particularly poorly when assessing aggressive behaviour for dogs. Also, participants were not better at assessing social interactions of humans compared to those of other species. We discuss what mechanism humans use to assess social situations and to what extent this skill can also be found in other social species.

## Introduction

A typical situation on a dog walk is that while two dogs are interacting, their owners like to interpret the behavior demonstrated by their dogs in terms of meaning and function. The owners often disagree with each other, as one may see a playful interaction whereas the other perceives it as rather aggressive. Even extensively trained humans, like behavioural scientists who observe human or animal interactions professionally, sometimes have difficulty to define and agree about the context in which a social interaction takes place (e.g. playful or aggressive) and then also understanding the meaning of behavioral signals [1, 2].

In order to ensure an optimal reaction in a given situation, however, it may be more important to predict the outcome of a situation rather than to define its context, per se. Using the example with the two dogs, determining to what extent the interaction is playful or aggressive is less critical than predicting whether or not the dogs are going to attack and harm each other in the next seconds. To make reliable predictions about future events and to minimize the costs of surprise, humans apply knowledge acquired during previous experiences to new situations (see [3, 4]). As accurate predictions usually lead to appropriate action choices (e.g. avoid risks or identify and react to danger, recognize beneficial chances or reflect mistakes, see [4–6], they have a beneficial effect on survival. In other words, predicting the outcome of a situation is highly adaptive.

Predictions about others’ intentional mental states and future actions are crucial during social interactions. To attribute intentional states to others, humans need to understand actions as being goal-directed. This ability, which is also important to predict outcomes of actions, emerges at a young age. Children around the age of six months are already able to perform goal-directed gazing [7, 8], a skill which continues to develop over the years. For example, Cannon & Woodward (2012) found that, in their first year, infants are already developing an understanding of goal-directed actions to make online predictions of others’ next actions [9]. They look predictively to an object that another person had previously reached, even when the object’s location had changed. For adults it was shown that within complex scenes, viewers tend to focus their attention on the most meaningful and task-relevant information [10], represented by a predictive gazing pattern (e.g. predicting the bounce point of a tennis match with direct fixation [11]). These findings suggest that human subjects rely more on predictive gazing (by producing eye movements similar to those produced when performing the task) than on reactive gazing (i.e. analysing the action) when observing a task [12], showing how important predictions of actions are in everyday life. However, so far most studies have focused on how humans predict the outcome of complex social interactions among members of their own species (but see [13]). But it would also be highly adaptive to predict the behaviour of other species, in particular when there is a risk of injury (see also dog example above).

While this gap in the literature still exists, much is known about how humans define the social context of a given situation and from it infer the meaning of the behaviours demonstrated by the subjects involved in the interaction. A typical approach for behavioural scientists is to establish ethograms by coding a huge number of interactions and their outcomes to interpret behaviors [14]. When those ethograms are established for a given animal, it is possible to identify social contexts by recognizing typical behaviours such as gestures. For example, thorough research has made it possible for experts to unambiguously recognize playful contexts in chimpanzees by detecting typical play gestures [15–17], affiliative contexts in ravens marked by the offering of non-edible items [18] and playful contexts in dogs characterised by their typical play bow [19].

However, some types of interactions are more difficult than others for non-experts to assign context to. Whereas it seems generally easy for humans to interpret a situation based on auditory signals in primates [20] and in dogs [21], the picture is much more complex for visual signals.

For example, most people are quite unskilled in interpreting the emotional states of chimpanzees [22], likely because chimpanzees have facial expressions that functionally differ from those of humans, despite the obvious physical similarities [23, 24]. Also, humans generally have problems interpreting the body language of dogs [22, 25–31]. Such interpretive decisions also depend on the kind of context. For example, Amici and colleagues (2019) found that six-year-old kids performed above chance level in interpreting dog emotions. Specifically, kids were better at detecting aggressive expressions than fearful and sad expressions. Indeed, from an evolutionary point of view, humans should predict aggressive outcomes better than playful and neutral ones (e.g. [4, 6]), because knowing when another individual of any species is aggressive might provide a clear selective advantage as one can react accordingly and avoid a potentially deadly encounter.

But even within the human species, we cannot be sure that we all interpret the emotional signals of the individuals involved in a situation the same way. Whereas it has long been assumed that human emotions are universal [32], more recent studies have shown that there are huge cultural differences in how emotions are expressed, interpreted, and labelled [33–38].

Overall, these studies provide evidence that humans can selectively attend to the emotional context of a social situation in order to assess it and make predictions about possible future outcomes. However, results from the above-mentioned studies do not allow reliable conclusions about our ability to predict intra- and interspecific social interactions in dynamic natural settings to be drawn. In the current study, we therefore aimed at testing the ability of healthy adult participants to assess various social situations between members of three species. For this purpose, we presented short video clips of three different real-life interactions (aggressive, neutral and playful) of dyads of dogs, monkeys (macaques) and human children. We then asked participants to either categorize the context of the situation–i.e. whether it was aggressive, neutral or playful–or to predict the outcome of the observed interactions. Dogs were chosen to assess interspecific interactions because they have been living closely with humans for up to 30,000 years and have undergone a special domestication process [39]. Macaques were chosen because of their relatedness as a primate species.

We hypothesized that humans would perform above chance level in predicting the outcome of social interactions across all species, as this improves the ability to react accordingly and economizes cognitive effort, which is beneficial for survival. Secondly, we hypothesized that participants would perform better at predicting the outcome of an interaction compared to categorizing the context, as the first task is more important for survival and therefore more adaptive than the second. Thirdly, we hypothesized that performance in assessing social interactions would vary depending on context. In particular, we expected that participants would perform better at rating the context and predicting the outcome of aggressive than they would for playful and neutral situations, as this is highly adaptive. Finally, we hypothesized that humans would be better at assessing social interactions between members of their own species due to inherent intra-species advantage, i.e. that we interact more frequently with other humans than we do with other species.

## Methods

### Subjects

We tested 92 participants (30 men and 62 women) ranging from 19 to 73 years of age (mean 31.4 years). Participants were randomly assigned to one of the two experimental tasks, i.e. either classifying the context or predicting the outcome of the video-clips. This resulted in two groups of 46 participants. Testing took place in different locations, mostly at the Friedrich Schiller University and at the MPI for the Science of Human History in Jena, but sometimes also in private places. One experimenter was always present to observe the procedure and answer any possible questions.

The study was approved by the ethical committee of the University of Jena and of the Max Planck Society and was carried out in accordance with the relevant guidelines and regulations. Participants took part on a completely voluntarily base and were not rewarded for taking part in the experiments. Written informed consent was obtained from all subjects prior the start of the experiment. Information about the precise aims of the study was provided to the participant only after the completion of the experiment. In case participants were interested, they were then allowed to watch the long version of the video clips, including the outcome (see below).

### Stimuli

In total, 54 video clips were prepared– 18 for each of the study species: dogs, monkeys and human children. Each of the clips contained a natural non-verbal interaction between 2 conspecifics (in a few clips a 3^rd^ conspecific was present, in which case the participants were instructed to focus their attention on the two relevant conspecifics). The total number of clips was split into two comparable sets containing 27 clips each.

The clips were selected by three researchers with previous extensive experience working with dogs, monkeys and humans (JB, LSO, FA). We defined 3 contexts: a neutral context (Neutral; i.e. no social interaction between the partners, e.g. two monkeys eating apples next to each other), an aggressive context (Aggressive; i.e. a negative interaction with species-typical aggressive or agonistic behaviour between the partners, e.g. a dog defending a toy) and a playful context (Playful; i.e. a positive interaction with species-typical play behaviour, e.g. children playing hide and seek). Moreover, three further experts for each species, blind to the aims of the study, categorized the context of each clip independently and agreed with the categorization of the authors (i.e. 83% agreement across species and clips).

Clips of dogs included different breeds recorded while freely interacting in gardens or dog parks. Clips of children included pre-linguistic children as well as pre-teens (1–10 years old). Clips of macaques included free-ranging Barbary macaques (*Macaca sylvanus*) housed at Kintzheim in France, as recorded during natural interactions in their group. The footage included vocalizations, but no verbal interactions (except for single words in the case of recording of human children) to make them comparable between humans and non-human animals. The video material was provided by the authors of the study and by volunteers who gave their permission to use the footages for scientific purposes. (The parents of the children pictured in the Video Clip Examples have provided written informed consent as outlined in PLOS consent form to publish their image alongside the manuscript).

We prepared 54 clips in total: six clips for each of the three contexts for each of the three species (6 clips x 3 contexts x 3 species). The video clips consisted of two parts, with only the first shown to participants. The first part of the clip, lasting 2- to 5-seconds, included information about the nature of the following interactions, including social cues like facial expressions and body postures. The clip was interrupted 10 frames before a social interaction took place (see S1 File and S1–S9 Videos). Thus, it could end in one of three different ways:

- Aggressive: one of the two individuals made an aggressive signal/action toward the other (e.g. stiff body posture in dogs, open mouth in monkeys, suddenly moving toward the partner in children);
- Playful: one of the two individuals made a playful signal/action toward the other (e.g. playful bow in dogs, playful face in monkeys, smile in children);
- Neutral: no obvious/apparent cue/action was displayed.

The second part of the clip, which was not shown to participants and lasted up to 17 s, depicted the aggressive, playful or neutral interaction and its outcome.

The experimenters also prepared sentences that described plausible outcomes of the video clips. These sentences stated how the interaction could continue, e.g. (a) one individual attacks the other one, (b) both individuals interact playfully with each other or (c) both individuals continue what they have done before without further interaction (for details, see also S1 File). Whenever possible, each of these three prepared sentences illustrated one of the contexts, i.e. one possibility was playful, one neutral and one aggressive.

### Procedure

Each participant was randomly assigned to one of two pre-determined groups and presented with 27 clips. Before the test started, the experimenter explained the basic procedures of the test and handed out a coding sheet.

During the test, participants were presented with a PowerPoint presentation consisting of one video clip per slide, which they could watch a maximum of two times. One group was tasked with classifying the context of the clip (i.e. Neutral, Aggressive or Playful). The other group was tasked with predicting the outcome of the situation. In that case, each slide with a clip was followed by a slide with sentences prepared by the experimenters describing the three possible outcomes (see above). Participants had to select one and note their responses on their coding sheet. It took participants 20–30 minutes to watch all clips and to complete the tasks.

### Design and coding

Each participant was presented with 27 video clips (3 species x 3 contexts x 3 clips per context) in randomized order. As mentioned above, each participant was assigned randomly to one of two pre-determined groups—participants of group 1 classified the context and participants of group 2 selected the outcome. Half of the participants in each group were tested with one prepared stimulus set, and the other half was tested with the other stimulus set, to exclude possible effects of the clips. After completing their main task participants were tested in the other task as well. Thus, in the context group participants predicted also the outcome, while in the outcome group participants also classified the context. However, as the two tasks are not independent of each other, signaled by the various significant effects of task-order and its interaction with the other variables, here we consider the two participant groups independently of each other and only present the analysis of the task they performed first (for additional information, see S1 File).

For the first group we scored Context Choice (i.e. which context was selected: Playful, Neutral or Aggressive) and Context Correct (whether the selected context was correct according to our classification). For the second group we scored Outcome Choice (i.e. which outcome was selected from the three possibilities) and Outcome Correct (whether the selected outcome was correct).

### Statistical analyses

We averaged each participant in group 1’s performance at categorizing the context for each for species and context, and each member of group 2’s performance at predicting the outcome for each for species and context.

The data was analysed with an ANOVA with species (3) and context (3) as within-subject and type of task (2, context or outcome) as between subject factors. Post-hoc tests were performed using Bonferroni correction. To test if the results are significantly different from chance (33%) we used a one-sample t-test. To correlate the performance of participants we used Pearson Correlation.

## Results

**Fig 1:**
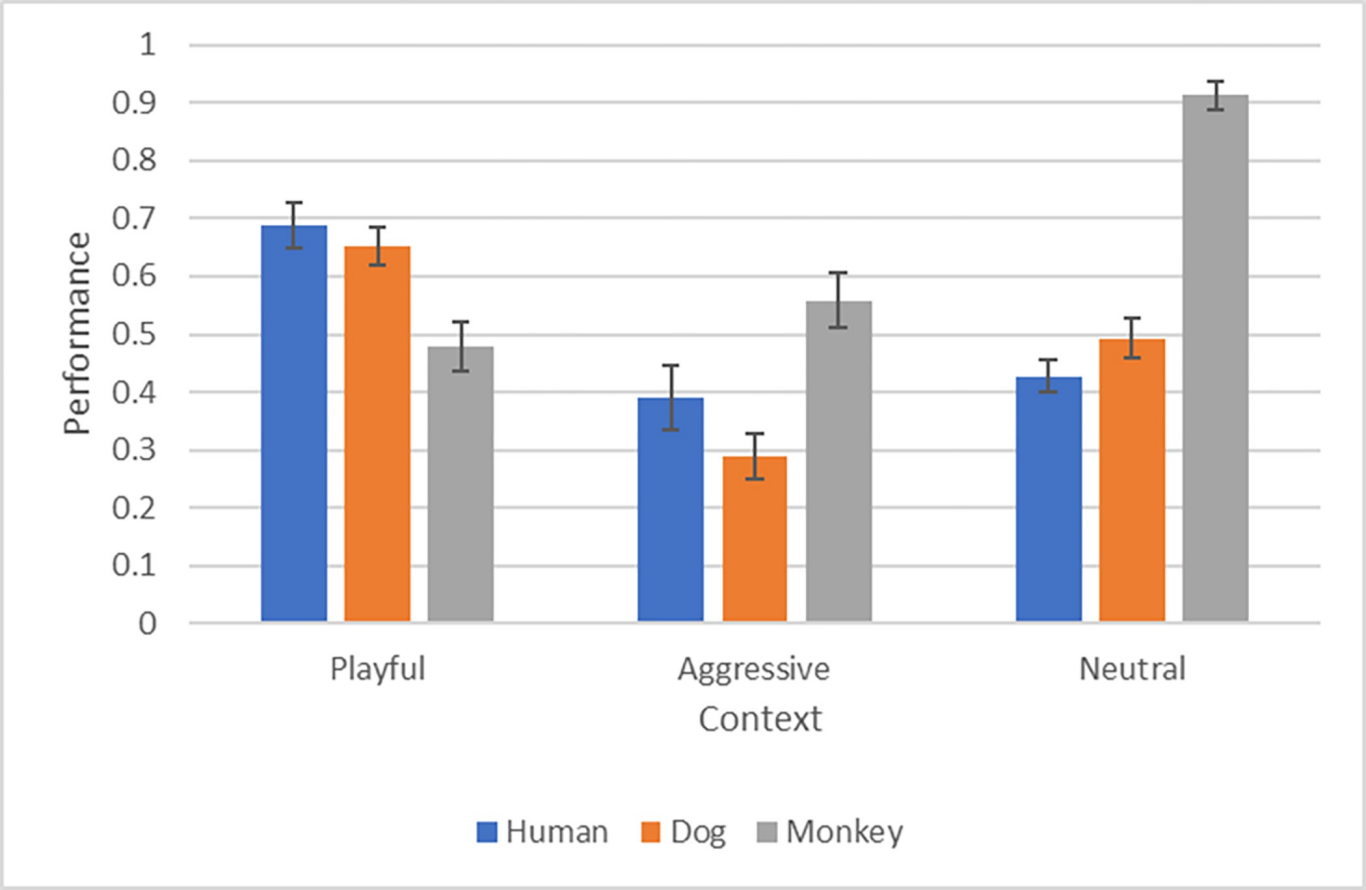
Depicts the average correct responses for the context identifications for each species and context in group 1.

**Fig 2:**
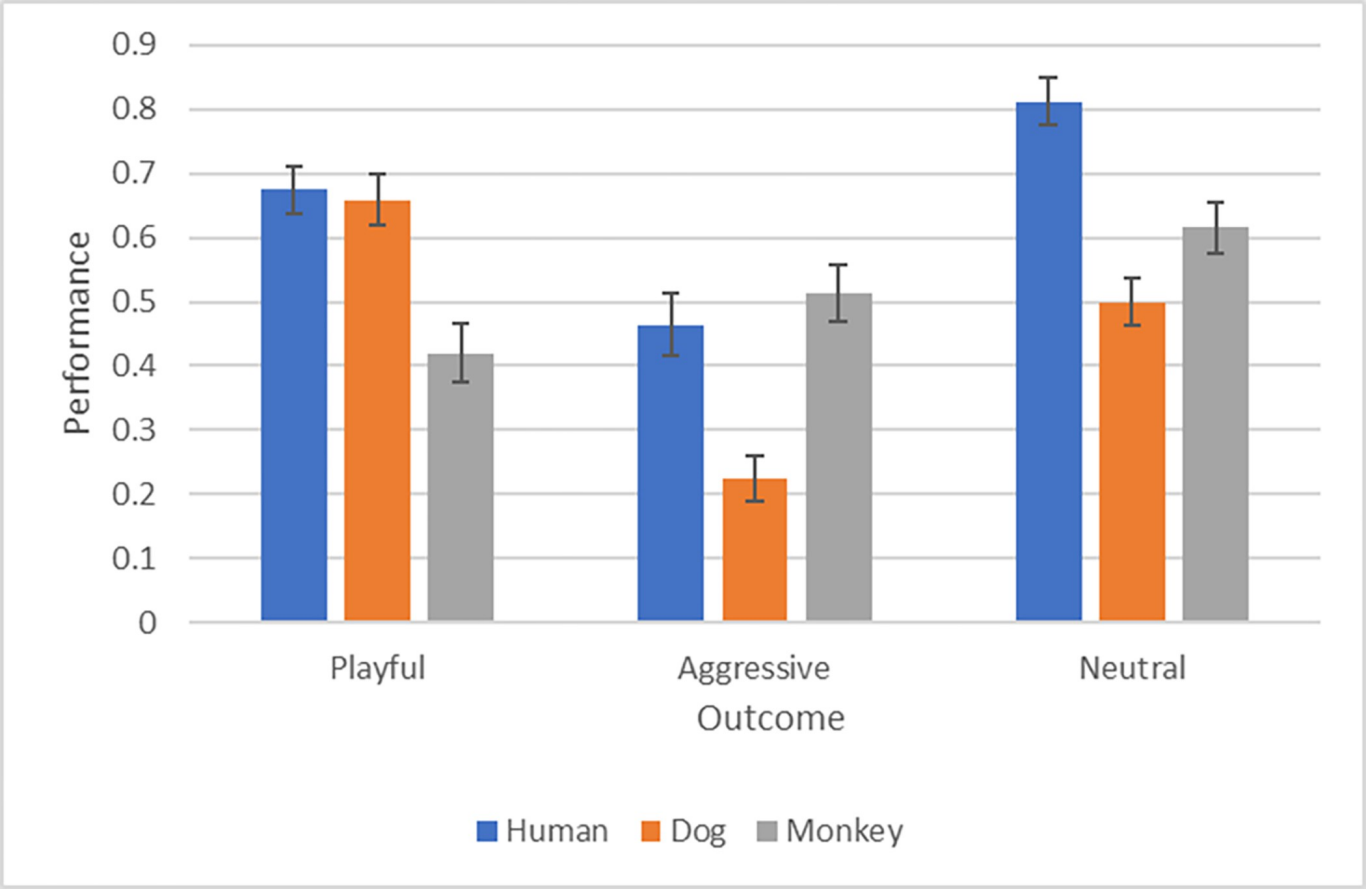
Depicts the average performance of the participants for the estimation of the future outcome of the interactions for each species and context in group 2.

Fig 1 presents the average context categorization scores and Fig 2 presents the average predictions of outcomes.

Participants performed similarly at predicting the outcome and categorizing the context (main effect of type of task: F(1, 90) = 8.70e-4, p = 0.977; η^2^ = 9.67e-6). However, there was a significant 3-way interaction of species, context and type of task (F(4,360) = 11.160, p < .001; η^2^ = 0.110), indicating that the performance of participants in the two groups differed depending on species and outcome. Furthermore, there was a significant interaction between species and type of task, indicating that participants in the two groups showed performed differently depending on the species (F(2, 180) = 21.40, p < .001; η^2^ = 0.192). However, context did not impact participants’ performance, as there was no significant interaction between type of task and context (F(2,180) = 0.943, p = 0.392; η^2^ = 0.110). Moreover, we found a significant interaction between species and context (F(4,360) = 29.40, p < .001; η^2^ = 0.246), demonstrating that participants performed differently for the three species and this effect depended on the context. Finally, performance was different for the three species (main effect of species: F(2,180) = 17.44, p < .001; η^2^ = 0.162), with all participants performing better with primates than dogs. Performance was also different for the three main contexts (main effect of context: F(2,180) = 66.51, p < .001; η^2^ = 0.425).

For the comparisons against chance level and the pairwise comparison we first consider the two types of task (tested in the groups) separately. Then we present the relevant comparisons between groups.

### Context decisions

Participants identified the context of the social interaction in the video clips better than chance level for most cases (P<0.002 for every comparison), with the exception of Aggressive interactions in dogs and in humans.

The Playful context was categorized very well in about 70% of trials with human and dog interactions, and monkeys interactions were categorized significantly worse than those between humans (Playful: human vs monkey t(45) = 3.95, p = 0.013). For the Aggressive Interactions, participants correctly categorized the context for monkeys more often than for dogs (Aggressive: dog vs monkey t(45) = -5.05, p<0.001). Similarly, for Neutral outcomes the context was categorized better for monkeys than for dogs and humans (Neutral: dogs vs monkeys t(45) = -7.91, p<0.001; humans vs monkeys t(45) = -9.14, p<0.001).

Within the species we found the following results: for dogs the context was categorized worst in the Aggressive context, while Playful interactions and Neutral interactions were categorized significantly better (Aggressive vs Playful t(45) = 6.91, p<0.001; Aggressive vs Neutral t(45) = -3.87, p = 0.019). For humans, the Playful context was categorized better than Neutral and Aggressive (Playful vs Neutral t(45) = 4.98, p<0.001, Playful vs Aggressive t(45) = 6.95, p<0.001). For monkeys, the pattern was different: context was categorized best in Neutral situations compared to the other two contexts (Neutral vs Aggressive t(91) = -6.77, p<0.001, Neutral vs Playful t(45) = -8.29, p<0.001). All other relevant comparisons were not significant.

Regarding individual performance of participants, we did not find correlations in the performance in rating the context between all three species (N = 46; P> = 0.215). For the correlation between contexts there was only one significant positive correlation between the playful and neutral context (r = 0.31, P = 0.039), indicating that participants that performed well in rating the context for playful interaction were also good in rating neutral interactions. The other two correlations were not significant (N = 46; P> = 0.387).

### Outcome decisions

Participants could predict the outcome of interactions significantly better than chance level for nearly all cases (P<0.010 for every comparison), with two exceptions. Participant ratings of Playful interactions in monkeys exhibited a strong trend (p = 0.054), while participant predictions of outcomes for Aggressive Interactions in dogs were significantly below chance level (p = 0.005).

In the Playful context, participants predicted the outcome significantly better for dogs and humans than for monkeys (dogs vs monkeys t(45) = 4.50, p = 0.001; humans vs monkeys t(45) = 4.77, p<0.001). In contrast, in the Aggressive context the outcomes were predicted significantly better for primates (i.e. humans and monkeys) than for dogs (Aggressive: humans vs dogs t(45) = 4.50, p = 0.001; monkeys vs dogs t(45) = -5.45, p<0.001). In the Neutral context, the outcome was predicted best for the humans compared to the other two species (Neutral: humans vs dogs t(45) = 5.86, p = 0.009; humans vs monkeys t(45) = 3.68, p = 0.038).

Within the species we found the following results: Participants predicted the outcome of a dog interaction in an Aggressive context compared to the Playful context and the Neutral context (Aggressive vs Playful t(45) = 8.29, p<0.001, Aggressive vs Neutral t(45) = -5.25, p<0.001). For humans there was the same pattern: participants predicted the outcome of an interaction worse in an Aggressive context compared to the Playful context and the Neutral context (Aggressive vs Neutral t(45) = -6.64, p<0.001; Aggressive vs Playful t(45) = 4.00, p = 0.011). For monkeys participants predicted the outcome better in the Neutral context compared to the Playful context (Neutral vs Playful t(45) = -3.73, p = 0.032). All other relevant comparisons were not significant.

### Comparison between context and outcome decisions

In this Post-hoc analysis we compared performance in the two tasks (group 1 categorizing contexts and group 2 predicting outcomes) for the same contexts and species, as there was a 3-way-interaction of species, context and type of task (see above). There were only significant results for the Neutral context. In particular, participants from group 1 were significantly better at categorizing the context of monkey interactions than participants of group 2 were at predicting the outcome (Neutral t(45) = 5.26, p<0.001). In contrast, participants of group 2 were significantly better at predicting the outcome of human interactions than participants of group 1 were at categorizing the context (Neutral t(45) = -6.80, p<0.001).

Regarding the individual performance we found positive correlations between predicting the outcome of social interactions between all three species (N = 46; kids and dogs: r = 0.35, P = 0.018; kids and monkeys: r = 0.56, P<0.001, monkeys and dogs: r = 0.48, P = 0.001), indicating that participants that were skilled predictors for one species, also performed well for the other two species. We also found positive correlations in predicting the outcome between all three contexts (N = 46; playful and aggressive: r = 0.56, P<0.001; playful and neutral: r = 0.43, P = 0.003, neutral and aggressive: r = 0.56, P<0.001), indicating that participants that were skilled predictors for one context, also performed well for the other contexts.

## Discussion

The participants in our study performed above chance level by predicting social situations in humans, dogs and monkeys. Presented with a choice of three outcomes, participants selected the correct one in 50–80% of interactions—as our first hypothesis predicted. Thus, for most of the nine situations participants were able to predict the outcome of the social interactions for all the species.

Yet, our data does not support our second hypothesis, as participants predicting the outcome of a social interaction in general did not perform better than participants who had categorized the context. Only in neutral human interactions did participants perform better at predicting outcomes than categorizing context. This shows that contexts can indeed be ambiguous, particularly for human interactions [see also 38]. It furthermore suggests that predicting the outcome of a given situation can sometimes be more constructive than simply categorizing its affective content. Surprisingly, there was one situation in which participants categorized the context *better* than predicting its outcome–i.e. in neutral situations with monkeys. Here, the performance in categorizing the context was very high–over 90%, whereas outcome predictions were about 60%. Thus, categorizing the context can be useful in general, but depending on the situation and the species, either categorizing or predicting may be more accurate. Obviously, these two tasks are not entirely independent of each other. When participants make a decision about the potential outcome of a situation, it is very likely that they also consider the social context, and when their task is to rate the context, they might also do it by partly taking into account its outcome. However, sometimes humans seem to consider context and outcome independently. In an everyday situation they might for example only speculate about the outcome without thinking how to define the context. It might also be important in what order the two tasks are tested experimentally (see S1 File). To investigate that question further, future studies should concentrate on one context and one species to exclude possible interaction effects between these factors.

Future studies should also take into account individual differences. Participants in our study that were especially skilful in predicting the outcome for one species or one context, also performed well for the other species or in the other contexts. Thus, there are people who are skilled predictors of social interactions in general–independent from species and context. Interestingly, we did not find such a correlation for rating the context, also indicating that contexts can indeed be ambiguous.

Our third hypothesis was that participants would be overall better at assessing aggressive situations than playful and neutral ones, independently of the species. We did not find evidence in our data to support this hypothesis. In contrast, participants performed poorly when assessing dogs`aggressive behaviour. In particular, they rated aggressive contexts among dogs at chance level, and they predicted outcomes below chance level. They also assessed aggressive interactions in dogs worse than playful and neutral ones. Thus, dogs`aggressive behaviour is not well-recognized. In addition, participants were unable to predict what could potentially occur next. Furthermore, other studies have shown that humans perform surprisingly poorly at detecting anxiety and aggression in dogs [28, 40, 41], but see also [22]. This is most likely the reason for the relative frequency of reported biting incidents [42, 43], as humans fail to notice dogs`displacement and appeasement behaviors before an attack [44]. A possible method of preventing severe biting incidents could entail that prospective dog owners are better educated about dog behaviour before adopting, as it has also been found that owning a dog does not improve the ability to assess dog behaviour [13, 45].

Interestingly, participants in our study also underestimated human aggressions. Participants performed below chance level at assessing the context, and also failed to reliably predict the outcome of aggressive interactions, performing worse than with playful and neutral contexts. It is possible that humans are biased to assume good intentions from other humans and from “man’s best friend”, sometimes preventing us from recognizing aggressive situations in these species.

In general, participant performance depended on both species and context. Thus, we have to reject our fourth hypothesis that humans will generally be better at assessing social interactions between members our own species than of other animal species. In the following, we consider the three contexts separately. For the playful context, participants assessed the situation better for dogs and humans than for monkeys. It is possible, that dogs`playful faces might be easier for humans to understand than the playful faces of other primates [46]. Also, other studies have found that humans more easily recognize positive dog emotions [13, 25, 28, 30, 31]. This could potentially be a consequence of co-domestication, meaning that through the process of domestication, humans became better at assessing when a dog is playing, and is therefore non-threatening. However, in other studies we found no evidence that humans have an innate tendency to understand dogs, in particular when it comes to aggressive interactions [13, 22], see also above.

Also, in contrast to our fourth hypothesis, participants were not better at assessing the social interactions of humans than of monkeys and dogs. (This was only the case for predicting the outcome in the neutral context.) Thus, we did not find a clear difference between species.

One could argue that our clips were not representative of species behaviors, or that the perceived intensity of the emotional contexts might differ across clips, or that some clips may not have enough context-specific information to be successfully assessed by participants. However, the clips were carefully selected with effort to eliminate biases towards any species. Indeed, each clip was assessed correctly either for context or outcome by at least 3 to 23 participants, indicating that the clips had sufficient context-specific information to be successfully assessed, but they were not self-explaining (see S1 File for details). For example, the performance was poor for every aggressive dog clip, indicating that the clips were comparable to each other and that humans are not very good in assessing aggressive dog interactions. We believe that the advantages of our decision to use clips of real social interactions outweigh the disadvantages of possibly different intensity of the emotional situations. Future studies should control for the apparent strength of the emotional context by using criteria; for example, whether there is physical contact between subjects or not, see [14].

One open question that emerges from the current study is how exactly humans`assessment of a social situation works. A recent study used eye-tracking of human participants while they watched video clips with naturalistic emotional expressions from dogs or humans. The study found that, although emotional states of dogs are more evident in bodily cues, humans focused mainly on the head of the subjects in the video [47]. Another study found that when only the facial expressions of primates and dogs are presented, participants predominantly attend to human eyes and animal mouths [48]. In particular, for judging great apes it would be helpful, if human observers would rather rely on vocalisations, as apes express aggressive intent mainly through vocalisations [49]. Future studies should use questionnaires to find out which features participants use to assess such a social interaction, and more importantly, to find ways to improve the performance of participants [13, 45, 46, 48].

The skill of perceiving and anticipating goal-directed actions does not seem to be uniquely human. Great apes, for example, are able to make online goal-based predictions about the actions of other individuals. Kano and Call (2014) tested great apes with an eye-tracking experiment in which the apes were familiarized with movie clips showing a human hand reaching to grasp one of two objects. In the test event the objects’ locations were swapped and the hand made an incomplete reach between the objects. The apes predictively looked at the familiarized goal object rather than the familiarized location when viewing the hand action in the test event. Thus, similar to human children, great apes make online goal-based predictions about the actions of other individuals [9, 50]. To compare the prediction ability of humans and non-human primates, Myowa-Yamakoshi, Scola and Hirata (2012) examined anticipatory eye movements in human infants, adults, and chimpanzees. The experiment showed that chimpanzees anticipate action goals in the same way as humans do, even though the chimpanzees used different methods to scan goal-directed actions [51]. Furthermore, there is evidence that dogs can perceive human actions as goal directed [52, 53]. Recent experiments have also used eye-tracking on animals to find out how they observe emotional expressions in other species. Dogs, for example, focus mainly on body signals when viewing emotional expressions of both conspecifics and humans [47]. Rhesus macaques explore human and monkey faces differently than chimpanzee and dog faces, indicating that their experience with the observed species might play a role [48]. Thus, animals are also able to assess natural social interactions in their own and other species, although they might sometimes use different mechanisms than humans.

To summarize, we show that humans perform above chance level at assessing natural social interactions in humans, in dogs and in monkeys–even without prior special experiences with the non-human species [13]. How well humans predict outcomes and categorize situations depends on the species and on the context. Humans are not better at assessing social interactions of their own species compared to other species. Surprisingly, participants perform particularly poorly when assessing dogs`aggressive behaviour. Thus, aggressive behaviours in dogs are not well recognized and the outcomes of aggressive interactions between dogs are hard for the average person to predict.

## Supporting information

S1 File Supplemental materials. (DOCX)

S1 Video Children play. (MP4)

S2 Video Children aggressive. (MP4)

S3 Video Children neutral. (MP4)

S4 Video Dog play. (MP4)

S5 Video Dog aggressive. (MP4)

S6 Video Dog neutral. (MP4)

S7 Video Monkey play. (MP4)

S8 Video Monkey aggressive. (MP4)

S9 Video Monkey neutral. (MP4)

S1 Data (XLSX)
