# Supplementary material for: Context and prediction matter for the interpretation of social interactions across species
Source: PLoS One. 2022 Dec 7;17(12):e0277783. doi: 10.1371/journal.pone.0277783 (PMC9728876; doi:10.1371/journal.pone.0277783)
Supplement: S1 File — (DOCX) [file pone.0277783.s001.docx]

**Context and prediction matter for the interpretation of social interactions across species**

Theresa Epperlein^1,2^, Gyula Kovacs^3^, Linda S. Oña^4^, Federica Amici^5,6^, Juliane Bräuer^1,2^

Each participant was assigned randomly to one of two pre-determined groups and was presented with 27 clips (presented in a PowerPoint presentation where each slide contained one clip). Before the test started, the experimenter explained the basic procedures of the test and handed out a coding sheet. The coding sheet contained the two tasks (either classifying the context or predicting the outcome of the video-clips), but in reverse orders. For the first group classifying the context of the clip was the first task, and predicting the outcome was the second task, for the second group it was the other way around. Each slide with a clip was followed by a slide that listed three choices of how the interaction could go on. Participants had to note down their responses on their coding sheet.

We found, that there were various order and interaction effects of the variables. In particular, in a 4-way ANOVA we found significant main effects for all 4 factors (context, outcome, species, context versus outcome, order), and also 9 out of 11 possible interaction effects were significant. In the current study we only considered the two groups independently of each other and only analysed the first task in each group (i.e. the context decision for the first group and the outcome decision for the second group) to avoid dependencies and order effects in our data-set.

Each clip was assessed correctly either for context or outcome by at least 3 to 23 participants (group 1 mean 12.50 participants SD 0.90; group 2 mean 12.48 participants SD 0.96).

Table S1: All used clips for the study with possible outcomes and actual outcomes (bold), and the numbers of participants (N), assessing the clips correctly.

| **Clip Dogs** | **A** | **B** | **C** | N  Context  correct | N  Outcome  Correct |
| --- | --- | --- | --- | --- | --- |
| 1 Aggressive Dog  **B7G3*** | Start playing with each other | **Nana (black) leaves and the other dog takes the toy** | Nana keeps the toy | 8 | 3 |
| 2 Aggressive Dog  **OAHX** | Both start playing with the toy | Ambula (right) keeps the toy | **Ambula leaves, Barry takes toy** | 1 | 6 |
| 3 Aggressive Dog  **EVE5*** | Nana (black) takes the toy | **Nana leaves** | Fight between both dogs | 5 | 1 |
| 4 Aggressive Dog  **RIP1** | **Nana tries to catch Leloo (big)** | Nana keeps on ignoring | Start playing fetch | 12 | 10 |
| 5 Aggressive Dog  **REPB** | Male tries to mount female | Male gives up and moves away | **Female attacks male** | 11 | 9 |
| 6 Aggressive Dog  **PM2l** | **left dog takes toy and is successfull in defending** | Both stand still and Nothing happens | Dogs start playing fetch with the toy | 3 | 2 |
| 1 Play Dog  **NL02*** | **Rumble tumble play with growling** | Leloo (big) leaves | Fight between both dogs | 22 | 22 |
| 2 Play Dog  **OIOA** | Fight between both dogs | **Friendly Play** | Small dog leaves | 8 | 5 |
| 3 Play Dog  **Y7Q4*** | Both dogs leave in different directions | **Careful cuddle and surrounding each other** | Start fighting | 18 | 13 |
| 4 Play Dog  **IPXY*** | Nana (black/white) leaves | Fight between both dogs | **Friendly rumble tumble play** | 13 | 11 |
| 5 Play Dog  **FOX9** | **Friendly rumble tumble play** | Fight between both dogs | Paula (small) leaves | 23 | 22 |
| 6 Play Dog  **JQIY*** | Nana (black/white) leaves | **Friendly rumble tumble play** | Start fighting | 6 | 18 |
| 1 Neutral Dog  **CPIT*** | **No further interaction** | Black dog starts barking and the other dog leaves | Cuddle play | 4 | 19 |
| 2 Neutral Dog  **7W1W** | Black dog starts barking at the other dog | Cuddle play | **The gold coloured dog leaves** | 19 | 17 |
| 3 Neutral Dog  **U0GL** | Angus (schwarz) leaves | **Angus stays where he is and Kilo is sniffing at him** | Fight over the water bowl | 21 | 0 |
| 4 Neutral Dog  **N9HK** | **Lotte leaves (big)** | Cuddle play | Fighting | 3 | 12 |
| 5 Neutral Dog  **PDKO** | Cuddle play | Sherlock starts barking at Paula | **Sherlock (behind) leaves to smell** | 6 | 11 |
| 6 Neutral Dog  **E92A** | Short fight | **Max leaves (black)** | Cuddle play | 15 | 10 |
| **Clip Monkeys** | **A** | **B** | **C** |  |  |
| 1 Aggressive Monkey  **XH00** | **Big monkey chases little monkey** | Big monkey does nothing | Monkeys rumble | 12 | 16 |
| 2 Aggressive Monkey  **0DGW** | Monkey on the left approaches monkey on the right | Nothing happens | **Monkey on the right chases monkey on the left** | 16 | 14 |
| 3 Aggressive Monkey  **Z0DW** | Monkey on the right leaves | **Monkey on the left chases monkey on the right** | Monkeys rumble | 12 | 17 |
| 4 Aggressive Monkey  **B4SLl** | **Monkey in the back chases monkey in the front** | Monkey in the front approaches other monkey | Monkey in the back runs away | 18 | 6 |
| 5 Aggressive Monkey  **HKMLl** | Big monkey leaves | **Big monkey chases little monkey** | Monkeys rumble | 15 | 15 |
| 6 Aggressive Monkey  **A2GLl** | **Big monkey attacks little monkey** | Monkeys rumble | Nothing happens | 4 | 3 |
| 1 Play Monkey  **IT3F** | **Monkeys rumble** | Monkey on the left runs away | Nothing happens | 11 | 13 |
| 2 Play Monkey  **OITQ** | Monkey on the left bites other monkey | Monkey on the left leaves | **Monkeys rumble** | 8 | 11 |
| 3 Play Monkey  **VG9E** | Nothing happens | **Monkeys rumble** | Monkeys chase each other | 9 | 15 |
| 4 Play Monkey  **KGUKl** | **Monkeys rumble** | Monkey on the right delouses the other monkey | One monkey attacks the other one | 13 | 9 |
| 5 Play Monkey  **PL01l** | Monkey on the right leaves | Monkey on the left attacks other monkey | **Monkeys rumble** | 8 | 1 |
| 6 Play Monkey  **FP03l** | Monkey in the back attacks monkey in the front | **Short play** | Both keep staying on the tree | 17 | 9 |
| 1 Neutral Monkey  **7U9O** | **Nothing happens** | Monkey in the back steals apple | Both start rumbling | 23 | 23 |
| 2 Neutral Monkey  **X79M** | Monkey in the front runs away | Monkey in the back throws lettuce | **Monkey in the back drops lettuce** | 22 | 12 |
| 3 Neutral Monkey  **58FC** | Monkey on the right chases monkey on the left | **monkeys eat** | Monkey on the right screams | 22 | 22 |
| 4 Neutral Monkey  **EWFLl** | **Nothing happens** | Monkey on the left attacks monkey on the right | Monkey on the right invites other one to play | 22 | 0 |
| 5 Neutral Monkey  **NE01l** | **The monkeys remain seated** | Right monkey chases left monkey | Monkeys rumble | 16 | 6 |
| 6 Neutral Monkey  **NP02l** | Monkey on the right invites other monkey to play | Monkey on the left steals leaf from other monkey | **Nothing happens** | 21 | 22 |
| **Clip Children** | **A** | **B** | **C** |  |  |
| 1 Aggressive Children  **PF01*** | They start playing with each other | **The child (right) pushes the other one down to get the phone** | The child (left) keeps the phone | 9 | 17 |
| 2 Aggressive Children  **AA01*** | **Boy pulls bucket away from the girl and both fight** | Girl takes the bucket | They start playing with the bucket | 14 | 3 |
| 3 Aggressive Children  **7985** | The child in red turns around and leaves | Both start playing with the cups together | **The incoming child tries to take the cups away and the child in red moans** | 11 | 7 |
| 4 Aggressive Children  **4115** | Both start playing with the stick | **The baby turns over and both start crying** | The boy with the cap holds the baby | 6 | 10 |
| 5 Aggressive Children  **AA02*** | The boy leaves | The girl takes the bucket and starts playing | **The boy tries to pull the bucket away from the girl** | 4 | 20 |
| 6 Aggressive Children  **PF03*** | **Child (right) takes the phone and the other child starts screaming** | Child (left) takes the phone | Both start playing with the phone | 10 | 7 |
| 1 Play Children  **4142** | **Boy starts talking and playing with the baby** | Boy steals the pacifier from the baby | Boy starts playing on his own | 11 | 16 |
| 2 Play Children  **0686** | Fight between both children | **Start playing catch** | The boy with the red hair stays where he is | 17 | 2 |
| 3 Play Children  **0345** | The smaller child leaves | The bigger child pushes the other one | **Both start jumping and laughing** | 23 | 23 |
| 4 Play Children  **0129** | The girl leaves | The boy screams at the girl | **Both start laughing** | 8 | 8 |
| 5 Play Children  **4514** | The child in grey goes away | **The child with the striped sweater hugs the other child** | The child in grey starts crying | 17 | 21 |
| 6 Play Children  **KS01** | **Both keep playing and searching for the object** | The child in red leaves | Btart pushing each other away | 19 | 23 |
| 1 Neutral Children  **LF01** | The girl (left) takes the paper from the other girl | **Both keep folding** | Playing with each other’s paper | 3 | 18 |
| 2 Neutral Children  **LF02** | **The girl (left) plays on her own** | Both playing | The girl (right) takes the toy | 9 | 15 |
| 3 Neutral Children  **0690** | **The boy with the hood climbs up and nothing happens** | The boy with the red hair starts screaming | The boys start playing with each other on the slide | 6 | 16 |
| 4 Neutral Children  **4931** | Short fight about the food | Children start playing | **All keep on eating** | 16 | 20 |
| 5 Neutral Children  **0770** | The child on the right steals the cup | **Nothing happens** | The child on the right invites the other one to play | 23 | 20 |
| 6 Neutral Children  **4440** | **Both keep spinning around** | Start fighting | The girl wants to leave | 2 | 23 |
